# Dr. N Kumar

**Published:** 2010

**Authors:** P. Elangovan

**Affiliations:** Secretory IASSTD & AIDS


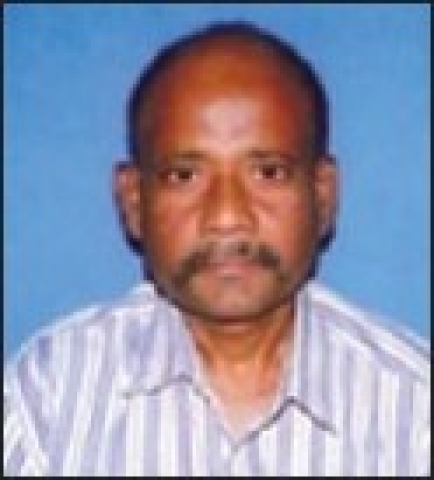


Dr. N. Kumar was born on 21/06/1952.

He completed his MBBS in April 1976 and went on to do DMRD which he completed in April 1982 and then D. V. in October 1984. He also did his MD in Venereology in the year October 1987.

He joined Tamilnadu Medical Service in 1984 and there he worked in various departments like Casualty, Forensic Medicine, Radiology and STD.

He was the recipient of Ranganathan Gold medal in 1986. He worked as a Professor in STD Dept at Tirunelveli Medical College in 2005. He joined Institute of Venereology as an Additional Professor in 2007 and he served as Director (i/c), Institute of Venereology from 2009.

He succumbed to his illness on 22/05/2010.

We pray that his soul rest in peace.

